# Disease-specific composite measures for psoriatic arthritis are highly responsive to a Janus kinase inhibitor treatment that targets multiple domains of disease

**DOI:** 10.1186/s13075-018-1739-0

**Published:** 2018-10-29

**Authors:** Philip Helliwell, Laura C. Coates, Oliver FitzGerald, Peter Nash, Enrique R. Soriano, M. Elaine Husni, Ming-Ann Hsu, Keith S. Kanik, Thijs Hendrikx, Joseph Wu, Elizabeth Kudlacz

**Affiliations:** 1 Leeds Institute of Rheumatic and Musculoskeletal Medicine, University of Leeds, 2nd Floor Chapel Allerton Hospital, Chapeltown Road, Leeds, LS7 4SA UK; 2 Nuffield Department of Orthopaedics Rheumatology and Musculoskeletal Sciences, University of Oxford, Windmill Road, Oxford, OX3 7LD UK; 3 Department of Rheumatology, St Vincent’s University Hospital, 196 Merrion Road, Elm Park, Dublin, D04 T6F4 Ireland; 4 Department of Medicine, University of Queensland, St Lucia, Brisbane, QLD 4072 Australia; 5 El Hospital Italiano se encuentra ubicado en Tte. Gral. Juan Domingo Perón 4190, C.A.B.A, Buenos Aires, Argentina; 6 Cleveland Clinic Lerner Research Institute, N building, 9620 Carnegie Avenue, Cleveland, OH 44106 USA; 7 Pfizer Inc, 280 Shennecossett Rd, Groton, CT 06340 USA; 8 Pfizer Inc, 500 Arcola Rd, Collegeville, PA 19426 USA

**Keywords:** Psoriatic arthritis, Assessment, PASDAS, DAPSA, CPDAI, DAS28–3(CRP)

## Abstract

**Background:**

The multiple disease domains affected in psoriatic arthritis (PsA) may make composite endpoints appropriate for assessing changes in disease activity over time. Tofacitinib is an oral Janus kinase inhibitor for the treatment of PsA. Data from two phase 3 studies of patients with PsA were used to evaluate the effect of tofacitinib on composite endpoints.

**Methods:**

Oral Psoriatic Arthritis triaL (OPAL) Broaden was a 12-month study of tumor necrosis factor inhibitor (TNFi)-naïve patients with an inadequate response to at least one conventional synthetic disease-modifying anti-rheumatic drug; OPAL Beyond was a 6-month study of patients with inadequate response to TNFi. Patients with active PsA received tofacitinib 5 or 10 mg doses twice daily (BID), adalimumab 40 mg subcutaneous injection once every 2 weeks (OPAL Broaden only), or placebo advancing at month 3 to tofacitinib 5 or 10 mg BID. The disease-specific composites were Psoriatic Arthritis Disease Activity Score (PASDAS), Disease Activity Index for Reactive Arthritis/Psoriatic Arthritis (DAPSA), and Composite Psoriatic Disease Activity Index (CPDAI). Change from baseline in composite endpoints was also assessed for minimal disease activity (MDA) responders versus non-responders.

**Results:**

Overall, 422 patients from OPAL Broaden and 394 patients from OPAL Beyond were treated. The mean changes from baseline to month 3 for tofacitinib 5 mg BID, tofacitinib 10 mg BID (standard error; effect size) were OPAL Broaden: PASDAS, −2.0 (0.14; 1.73), −2.4 (0.14; 2.4); DAPSA, −20.2 (1.72; 0.9), −24.4 (1.73; 1.23); and CPDAI, −2.9 (0.34; 1.03), −4.2 (0.36; 1.53); OPAL Beyond: PASDAS, −1.9 (0.14; 1.53), −2.1 (0.14; 1.84); DAPSA, −22.5 (1.67; 0.81), −21.0 (1.70; 0.84); and CPDAI, −3.3 (0.31; 1.41), −3.4 (0.31; 1.45). Greater changes from baseline to month 3 (*P* ≤0.05) were seen with both doses of tofacitinib versus placebo for all endpoints except CPDAI for tofacitinib 5 mg BID in OPAL Broaden. Effect sizes generally increased from 3 to 6 months. Mean changes from baseline were greater in MDA responders than MDA non-responders for all composite endpoints across all time points and treatments.

**Conclusions:**

This analysis suggests that disease-specific composite measures are appropriate for evaluating treatment efficacy on multiple disease domains in PsA.

**Trial registration:**

OPAL Broaden: ClinicalTrials.gov Identifier: NCT01877668, first posted June 12, 2013; OPAL Beyond: ClinicalTrials.gov Identifier: NCT01882439, first posted June 20, 2013.

**Electronic supplementary material:**

The online version of this article (10.1186/s13075-018-1739-0) contains supplementary material, which is available to authorized users.

## Background

Psoriatic arthritis (PsA) is a chronic, systemic, immune-mediated inflammatory disease with multiple disease manifestations, including peripheral arthritis, enthesitis, dactylitis, spondylitis, and psoriatic skin and nail disease [1–3]. Owing to the multiple diverse disease manifestations involved in PsA, the Group for Research and Assessment of Psoriasis and Psoriatic Arthritis (GRAPPA) bases its treatment recommendations on the domains affecting an individual [1]. Consequently, composite endpoints, which allow the assessment of multiple clinical outcomes in a single instrument, have been suggested to be particularly useful to assess changes in the multiple disease domains of PsA over time [3, 4]. Composite endpoints also have the potential to simplify statistical testing in clinical trials as a summary or total score is usually generated, thus requiring only a single hypothesis test, thereby avoiding issues with multiplicity and allowing for appropriate statistical power with relatively small numbers of patients [5].

A number of composite endpoints have been developed for PsA in order to assess multiple aspects of disease activity and identify patients who have achieved treatment targets of remission or minimal disease activity (MDA). Available instruments incorporate different types of assessments, including clinical (for example, tender and swollen joint counts [TJC and SJC]), laboratory (for example, C-reactive protein [CRP]), and patient-reported outcome (PRO) (for example, Health Assessment Questionnaire-Disability Index [HAQ-DI]) endpoints. Although there is no clear agreement on a standardized composite assessment approach that provides the optimal combination of individual variables [6], agreement has now been reached on a core domain set of variables that should be included [7].

Tofacitinib is an oral inhibitor of the Janus kinase (JAK) family for the treatment of PsA. Tofacitinib preferentially inhibits signaling via JAK3 or JAK1 (or both) with functional selectivity over JAK2 [8]. The efficacy and safety of tofacitinib 5 and 10 mg twice daily (BID) have been demonstrated in patients with PsA with an inadequate response to conventional synthetic disease-modifying anti-rheumatic drugs (csDMARDs) in Oral Psoriatic Arthritis triaL (OPAL) Broaden [9] and in patients with PsA who were tumor necrosis factor inhibitor (TNFi)-inadequate responders (IRs) in OPAL Beyond [10]. In both studies, tofacitinib had greater efficacy than placebo on the basis of the primary endpoints: a higher proportion of patients receiving tofacitinib than placebo achieved greater than or equal to 20% improvement according to the criteria of the American College of Rheumatology (ACR20 response) at month 3, and the mean change from baseline to month 3 in HAQ-DI score was greater in patients receiving tofacitinib versus placebo at month 3. In addition, between 21% and 26% of patients receiving tofacitinib and between 7% and 15% of patients receiving placebo had MDA responses at month 3 in OPAL Broaden and OPAL Beyond [9, 10]. This analysis evaluated the effect of tofacitinib on three disease-specific composite endpoints in patients with PsA by using data from the two placebo-controlled, double-blind, multicenter, global phase 3 studies of tofacitinib detailed above: OPAL Broaden and OPAL Beyond [9, 10].

## Methods

### Patients

Details of patient populations and study designs for both OPAL Broaden (A3921091; ClinicalTrials.gov Identifier: NCT01877668) and OPAL Beyond (A3921125; ClinicalTrials.gov Identifier: NCT01882439) have been published in detail [9, 10]. In brief, for inclusion in either OPAL Broaden or OPAL Beyond, patients were required to have active PsA with a duration of at least 6 months, to fulfill ClASsification criteria for Psoriatic ARthritis (CASPAR) at screening, and to have evidence of active arthritis with both a TJC and SJC of three or higher. Patients in OPAL Broaden had an inadequate response to at least one csDMARD and were TNFi-naïve, whereas patients in OPAL Beyond had an inadequate response to at least one TNFi. The primary endpoints in both studies were ACR20 response rate and change from baseline in HAQ-DI score at month 3.

### Study design

OPAL Broaden was a 12-month study in which patients were randomly assigned 2:2:2:1:1 to receive tofacitinib 5 mg BID, tofacitinib 10 mg BID, adalimumab 40 mg subcutaneous (SC) injection once every 2 weeks (Q2W), placebo advancing to tofacitinib 5 mg BID at month 3, or placebo advancing to tofacitinib 10 mg BID at month 3. OPAL Beyond was a 6-month study in which patients were randomly assigned 2:2:1:1 to receive tofacitinib 5 mg BID, tofacitinib 10 mg BID, placebo advancing to tofacitinib 5 mg BID at month 3, or placebo advancing to tofacitinib 10 mg BID at month 3. In both studies, patients also received one concomitant treatment with a stable dose of either methotrexate or another csDMARD (for example, sulfasalazine or leflunomide).

### Assessments

Three disease-specific composite endpoints are discussed in this analysis. The Psoriatic Arthritis Disease Activity Score (PASDAS) (score range of 0–10) includes the following components: patient’s global joint and skin assessment (visual analog scale; VAS [in millimeters]); physician’s global assessment of PsA (VAS [in millimeters]); SJC (66 joints) and TJC (68 joints); Leeds Enthesitis Index (LEI) score; tender dactylitic digit score; physical component summary (PCS) score of the 36-item short-form survey version 2 (SF-36v2 acute, norm-based scores); and CRP (in milligrams per liter) (Table 1) [11]. The Disease Activity Index for Reactive Arthritis/Psoriatic Arthritis (DAREA/DAPSA) (score range not defined; referred to as DAPSA herein) includes the components SJC (66 joints) and TJC (68 joints); patient’s global assessment of arthritis and patient’s pain assessment (both measured by VAS [in millimeters]); and CRP (in milligrams per liter) (Table 1) [6]. The Composite Psoriatic Disease Activity Index (CPDAI) (score range of 0–15) includes the components peripheral arthritis (SJC, TJC, and HAQ-DI); skin disease (Psoriasis Area and Severity Index [PASI] and Dermatology Life Quality Index [DLQI]); enthesitis (LEI score and HAQ-DI); dactylitis (number of digits and HAQ-DI); and spinal disease (Bath Ankylosing Spondylitis Disease Activity Index and Ankylosing Spondylitis Quality of Life [ASQoL]) (Table 1) [12]. For each of these composite endpoints, a higher score indicates higher disease activity. For comparison, a non-disease-specific composite outcome measure was also assessed: the three-component Disease Activity Score using 28 joints with CRP (DAS28–3 [CRP]; score range of 0–9.4, a higher score corresponds to worse symptoms) includes the components SJC (28 joints) and TJC (28 joints) and CRP (in milligrams per liter) (Table 1) [13].

**Table 1:** Components of the composite endpoints PASDAS, DAPSA, CPDAI, and DAS28–3(CRP)

	Skin manifestations	Enthesitis	Dactylitis	Joints	Axial	PROs
PASDAS [11]	✓	✓	✓	✓		✓
DAPSA [6]				✓		✓
CPDAI [12]	✓	✓	✓	✓	✓	✓
DAS28–3(CRP) [13]				✓		

*Abbreviations*: *CPDAI* Composite Psoriatic Disease Activity Index, *DAPSA* Disease Activity Index for Psoriatic Arthritis, *DAS28–3(CRP)* 3-component Disease Activity Score using 28 joints with C-reactive protein, *PASDAS* Psoriatic Arthritis Disease Activity Score, *PRO* patient-reported outcomes

### Statistical analysis

The full analysis set (FAS) comprised all patients who were randomly assigned to the study and received at least one dose of study medication. Changes from baseline analyses were based on a repeated measures model, without imputation for missing values in the FAS, with the fixed effects of treatment, visit, treatment-by-visit interaction, geographic location, and baseline value; an unstructured covariance matrix was used. For results up to month 3, patients randomly assigned to the two placebo sequences were combined into a single placebo group. The repeated measures model included data from all visits up to month 3 for the treatment groups of tofacitinib 5 mg BID, tofacitinib 10 mg BID, adalimumab 40 mg SC Q2W (OPAL Broaden only), and placebo. For results beyond month 3 to the end of study, the two placebo sequences were analyzed separately. The calculation of effect sizes and standardized response means for treatment groups of tofacitinib 5 mg BID, tofacitinib 10 mg BID, and adalimumab (OPAL Broaden only) at months 3, 6, and 12 (OPAL Broaden only at month 12) was based on patients with greater than or equal to 3% baseline psoriasis body surface area (BSA) in the FAS in order to permit comparison based on the same set of patients, with no missing values for any of the three disease-specific composite endpoints at baseline or months 3, 6, and 12 (OPAL Broaden only at month 12).

The effect size for a given composite endpoint at a time point was defined as (mean at baseline – mean at time point)/(standard deviation [SD] at baseline). The standardized response mean for a given composite endpoint at a time point was defined as (mean at baseline – mean at time point)/(SD of change from baseline at time point). Effect size and standardized response mean are unitless measures and are adjusted for the endpoints’ variability, which allows comparisons to be made. For both effect sizes and standardized response means, levels of responsiveness have been proposed as small (≥0.20 to <0.5), moderate (≥0.50 to <0.8), and large (≥0.80), respectively [3, 14].

In order to investigate the relative strength of the composite endpoints in predicting MDA response at a given time point, multiple logistic regression was used to model MDA response as a dependent variable and the mean changes from baseline of the three disease-specific composite endpoints at the same time point as predictors. The estimated slope coefficient from this regression model is the change in log-odds of MDA response resulting from a 1-unit increase in change from baseline of the composite endpoint. It represents the strength of association between the composite endpoint and MDA response and is standardized (STB, range unbounded) to adjust for the variability of the composite endpoint to permit comparison of their associations with MDA response. In order to compare the correlations of the three disease-specific composite endpoints with MDA response, another standardized measure related to STB above, called logistic pseudo partial correlation (denoted as R, range of −1 to 1), was also calculated [15]. A value of R closer to 1 or −1 indicates strong correlation, whereas a value of 0 indicates lack of correlation. This regression analysis was performed separately for months 3, 6, and 12 (OPAL Broaden only for month 12) and separately for tofacitinib 5 mg BID, 10 mg BID, and adalimumab 40 mg SC Q2W. These analyses included the same set of patients with baseline psoriasis BSA of greater than or equal to 3% in the FAS with no missing values for any of the three disease-specific composite endpoints and MDA at months 3, 6, and 12 (OPAL Broaden only at month 12). MDA was defined as any five of the following seven criteria being met: TJC ≤1, SJC ≤1, PASI score ≤1 or psoriasis BSA ≤3%, patient arthritis pain (VAS) ≤15 mm, patient’s global assessment of arthritis (VAS) ≤20 mm, HAQ-DI ≤0.5, tender entheseal points (using LEI) ≤1 [16].

The PASDAS response rate was calculated at months 3, 6, and 12 (OPAL Broaden only for month 12) as the percentage of patients who had a good response (defined as a PASDAS score of less than or equal to 3.2 and a decrease from baseline in PASDAS score of greater than or equal to 1.6 at the relevant time point for patients with baseline PASDAS score of greater than 3.2 in FAS) [17]. Non-responder imputation was applied, and a missing response was treated as non-response.

The derivation of the composite endpoints was pre-specified in the original study protocols and statistical analysis plans; except for analysis using a repeated measures model, all analyses were performed post hoc. *P* values are reported for comparisons with placebo in repeated measures model analyses and for testing slope coefficients in multiple logistic regression analyses without adjustment for multiplicity. The significance level was set at two-sided, less than or equal to 0.05.

## Results

### Patients

The FAS comprised 422 patients from OPAL Broaden and 394 patients from OPAL Beyond (Table 2). Demographics and baseline disease characteristics have been published previously [9, 10]. Baseline values for composite endpoints were generally similar across treatment groups and studies (Table 2).

**Table 2:** Composite endpoint scores in the OPAL Broaden and OPAL Beyond studies (FAS)

Mean composite endpoint scores (SD) [number of patients evaluable at time point]									
	OPAL Broaden (*N* = 422)					OPAL Beyond (*N* = 394)			
	Tofacitinib 5 mg BID *N* = 107	Tofacitinib 10 mg BID *N* = 104	Adalimumab 40 mg SC Q2W *N* = 106	Placebo - > tofacitinib 5 mg BID *N* = 52^a^	Placebo - > tofacitinib 10 mg BID *N* = 53^a^	Tofacitinib 5 mg BID *N* = 131	Tofacitinib 10 mg BID *N* = 132	Placebo - > tofacitinib 5 mg BID *N* = 66^a^	Placebo - > tofacitinib 10 mg BID *N* = 65^a^
PASDAS									
Baseline	6.03 (1.15) [105]	6.01 (1.06) [102]	5.92 (1.25) [106]	6.03 (1.15) [103]		6.09 (1.22) [124]	6.43 (1.21) [128]	5.97 (1.26) [128]	
Month 3	4.17 (1.69) [98]	3.70 (1.43) [102]	3.89 (1.57) [98]	4.97 (1.41) [100]		4.26 (1.71) [122]	4.24 (1.78) [114]	5.07 (1.86) [117]	
Month 6	3.63 (1.61) [99]	3.43 (1.45) [98]	3.41 (1.47) [98]	4.00 (1.49) [48]	3.69 (1.46) [47]	3.87 (1.72) [117]	3.97 (1.75) [109]	3.81 (1.68) [55]	3.60 (1.71) [55]
Month 12	3.29 (1.37) [95]	3.05 (1.22) [95]	3.20 (1.56) [93]	3.50 (1.40) [44]	3.15 (1.21) [43]	–	–	–	–
DAPSA									
Baseline	45.55 (20.33) [107]	43.69 (19.51) [104]	38.52 (18.17) [105]	43.81 (22.46) [105]		45.53 (23.51) [130]	51.54 (27.80) [132]	42.64 (22.99) [131]	
Month 3	26.08 (23.53) [101]	20.29 (18.86) [103]	21.44 (20.43) [99]	31.64 (23.64) [101]		24.32 (20.53) [123]	29.14 (25.21) [117]	33.69 (27.69) [117]	
Month 6	21.42 (21.71) [100]	16.86 (17.99) [99]	16.34 (18.63) [99]	19.93 (18.14) [48]	19.42 (21.17) [48]	20.69 (17.55) [123]	28.30 (28.92) [113]	21.12 (20.75) [56]	20.28 (20.94) [55]
Month 12	15.14 (14.29) [95]	13.21 (13.87) [96]	13.84 (15.50) [93]	15.60 (18.07) [44]	14.83 (14.84) [44]	–	–	–	–
CPDAI^b^									
Baseline	9.9 (2.39) [81]	10.0 (2.76) [68]	9.7 (2.84) [77]	9.9 (2.65) [81]		10.1 (2.58) [79]	10.7 (2.56) [79]	9.6 (2.86) [85]	
Month 3	7.4 (3.57) [77]	6.0 (3.01) [68]	6.9 (3.19) [75]	8.0 (2.95) [78]		7.1 (3.23) [70]	7.1 (3.33) [74]	8.0 (3.80) [73]	
Month 6	6.2 (3.39) [76]	5.1 (2.87) [66]	5.6 (3.30) [74]	6.4 (3.45) [39]	6.4 (3.36) [34]	6.3 (3.42) [75]	6.5 (3.11) [69]	6.3 (3.43) [32]	6.1 (3.39) [35]
Month 12	5.1 (2.96) [73]	4.4 (2.71) [65]	5.0 (3.19) [71]	4.9 (3.17) [36]	5.1 (2.63) [32]	–	–	–	–
DAS28–3(CRP)									
Baseline	4.56 (0.92) [107]	4.48 (0.97) [104]	4.38 (1.02) [106]	4.50 (1.04) [105]		4.51 (1.04) [131]	4.67 (1.17) [132]	4.40 (1.03) [131]	
Month 3	3.25 (1.26) [101]	2.94 (1.15) [103]	2.96 (1.15) [99]	3.85 (1.30) [101]		3.18 (1.22) [123]	3.41 (1.39) [118]	3.77 (1.32) [117]	
Month 6	2.90 (1.25) [100]	2.60 (1.09) [99]	2.62 (1.08) [99]	2.99 (1.23) [48]	2.86 (1.23) [48]	2.98 (1.21) [123]	3.29 (1.36) [113]	2.87 (1.17) [56]	2.95 (1.35) [55]
Month 12	2.64 (1.11) [95]	2.43 (0.99) [96]	2.44 (1.04) [93]	2.60 (1.20) [44]	2.52 (1.05) [44]	–	–	–	–

^a^For baseline and month 3 visits, patients from the two placebo sequences were combined into a single placebo group

^b^Only patients with psoriasis body surface area (BSA) of greater than or equal to 3% were included

*Abbreviations*: *BID* twice daily, *CPDAI* Composite Psoriatic Disease Activity Index, *DAPSA* Disease Activity Index for Psoriatic Arthritis, *DAS28–3(CRP)* 3-component Disease Activity Score using 28 joints with C-reactive protein, *FAS* full analysis set, *N* number of patients in the full analysis set, *OPAL* Oral Psoriatic Arthritis triaL, *PASDAS* Psoriatic Arthritis Disease Activity Score, *Q2W* once every 2 weeks, *SC* subcutaneous, *SD* standard deviation

### Composite endpoint outcomes

#### PASDAS

**Fig. 1:**
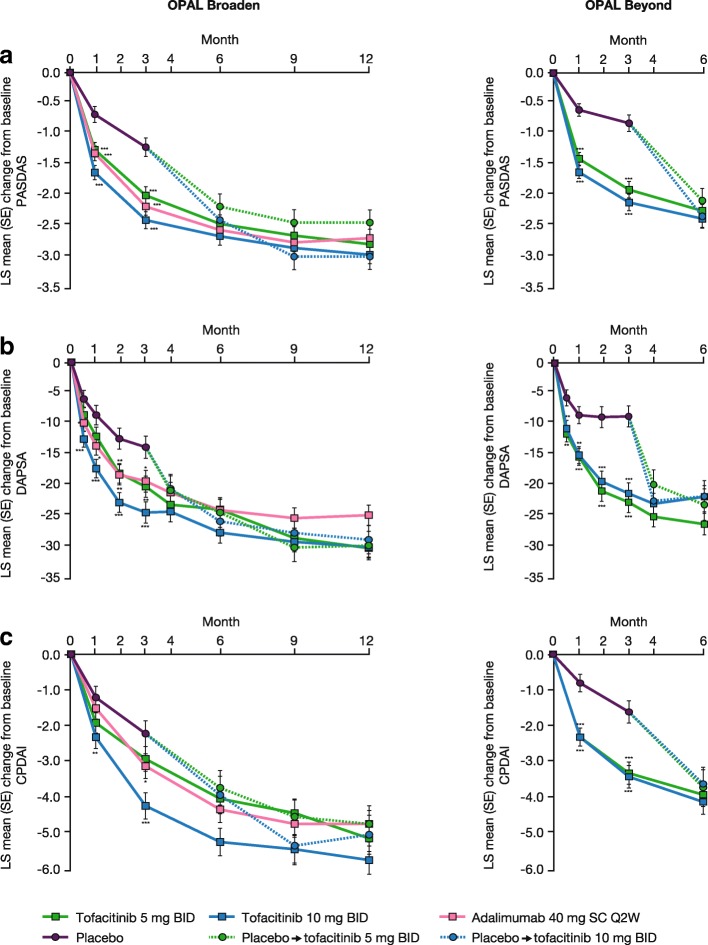
LS mean change from baseline in (**a**) PASDAS, (**b**) DAPSA, and (**c**) CPDAI. For complete data, see Additional file 1: Table S1. *P ≤0.05, **P <0.01, ***P <0.001 versus placebo. Abbreviations: *BID* twice daily, *CPDAI* Composite Psoriatic Disease Activity Index, *DAPSA* Disease Activity Index for Psoriatic Arthritis, *LS* least squares, *OPAL* Oral Psoriatic Arthritis triaL, *PASDAS* Psoriatic Arthritis Disease Activity Score, *Q2W* once every 2 weeks, *SC* subcutaneous, *SE* standard error

At baseline, mean PASDAS scores ranged from 5.92 to 6.43 across treatment groups in the OPAL Broaden and OPAL Beyond studies (Table 2). There were significantly greater improvements, as indicated by the least squares (LS) mean change from baseline in PASDAS score, with tofacitinib 5 and 10 mg BID versus placebo as early as month 1, continuing to month 3. Following advancement from placebo to tofacitinib treatments at month 3, patients showed similar improvements in disease activity at month 6 to those observed in patients receiving tofacitinib 5 or 10 mg BID throughout (Additional file 1: Table S1 and Fig. 1a); this improvement was maintained until the end of the 12-month OPAL Broaden study (Table 2 and Fig. 1a) and was larger in patients in the placebo advancing to tofacitinib 10 mg BID group than the placebo advancing to tofacitinib 5 mg BID group. In OPAL Broaden, the mean absolute PASDAS scores decreased to 3.29 and 3.05 with tofacitinib 5 and 10 mg BID, respectively, at month 12, and in OPAL Beyond the mean PASDAS scores at month 6 were 3.87 and 3.97 with tofacitinib 5 and 10 mg BID, respectively (Table 2). In OPAL Broaden, decreases from baseline in PASDAS scores were also observed in patients receiving adalimumab 40 mg SC Q2W from month 1 and were maintained to month 12 (Additional file 1: Table S1 and Fig. 1a).

### PASDAS response rates

**Fig. 2:**
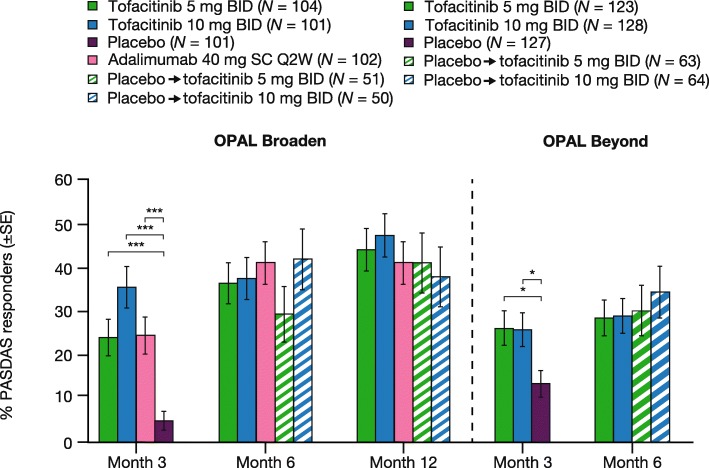
PASDAS response rates for patients with baseline PASDAS >3.2 (FAS). *P≤0.05, ***P<0.001 versus placebo. PASDAS response was defined as the percentage of patients who had a PASDAS score ≤3.2 and a decrease from baseline in PASDAS score ≥1.6 at the relevant time point. A missing PASDAS response at a given time point was imputed as non-response. Abbreviations: *BID* twice daily, *FAS* full analysis set, *N* number of patients with baseline PASDAS >3.2 in the FAS, *OPAL* Oral Psoriatic Arthritis triaL, *PASDAS* Psoriatic Arthritis Disease Activity Score, *Q2W* once every 2 weeks, *SC* subcutaneous, *SE* standard error

The percentage of PASDAS responders increased with time on treatment in both studies and with both doses of tofacitinib (Fig. 2). A similar percentage of patients initially randomly assigned to receive placebo were PASDAS responders compared with those patients receiving tofacitinib throughout at month 6 in OPAL Beyond and month 12 in OPAL Broaden, following advancement from placebo to active treatment with tofacitinib at month 3 (Fig. 2).

#### DAPSA

At baseline, mean DAPSA scores in the two studies ranged from 38.52 to 51.54 across treatment groups (Table 2). In OPAL Broaden, significantly greater LS mean changes from baseline in the DAPSA score (indicating improvement) were observed versus placebo for tofacitinib 10 mg BID from week 2 and for tofacitinib 5 mg BID from month 2, and differences were maintained to month 3 for both doses (Fig. 1b). In OPAL Beyond, significantly greater improvements, as indicated by LS mean changes from baseline in the DAPSA score, were observed with both doses of tofacitinib versus placebo from week 2 throughout the placebo-controlled period (Fig. 1b). The LS mean changes from baseline in the DAPSA score showed further decreases to month 6 with tofacitinib treatment in both studies (Fig. 1b), and the improvement was maintained to month 12 in OPAL Broaden (Additional file 1: Table S1 and Fig. 1b). At month 12 in OPAL Broaden, the mean absolute DAPSA scores were 15.14 and 13.21 with tofacitinib 5 and 10 mg BID, respectively, whereas in OPAL Beyond they decreased to 20.69 and 28.30 at month 6 with tofacitinib 5 and 10 mg BID, respectively (Table 2). At month 6 in both studies and month 12 in OPAL Broaden, following advancement from placebo to tofacitinib at month 3, patients showed similar improvements in DAPSA scores to patients receiving tofacitinib throughout (Table 2 and Fig. 1b). Patients receiving adalimumab 40 mg SC Q2W showed decreases from baseline in DAPSA scores throughout both OPAL Broaden and OPAL Beyond (Table 2 and Fig. 1b).

#### CPDAI

At baseline, mean CPDAI scores ranged from 9.6 to 10.7 across studies and treatment groups (Table 2). For CPDAI, significant improvements in LS mean change from baseline were seen for tofacitinib 10 mg BID but not tofacitinib 5 mg BID versus placebo at months 1 and 3 in OPAL Broaden (Fig. 1c). Significant improvements in LS mean change from baseline in CPDAI score versus placebo at months 1 and 3 were reported for patients receiving tofacitinib 5 and 10 mg BID in OPAL Beyond (Fig. 1c). Decreases in LS mean change from baseline in CPDAI score with both doses of tofacitinib were maintained at months 6 and 12 (Fig. 1c). In OPAL Broaden, the mean absolute CPDAI scores with tofacitinib 5 and 10 mg BID at month 12 were 5.1 and 4.4, respectively, and in OPAL Beyond they were 6.3 and 6.5, respectively, at month 6 (Table 2). Patients who advanced from placebo to tofacitinib treatments at month 3 showed similar improvements in CPDAI absolute scores and LS mean change from baseline in CPDAI at month 12 in OPAL Broaden and month 6 in OPAL Beyond to patients receiving tofacitinib 5 or 10 mg BID throughout (Table 2 and Fig. 1c). In OPAL Broaden, patients receiving adalimumab 40 mg SC Q2W showed improvements from baseline in CPDAI scores from month 1 to 12 (Table 2 and Fig. 1c).

#### DAS28–3(CRP)

For the non-disease-specific comparator included here, mean DAS28–3(CRP) at baseline was 4.38 to 4.67 across studies and treatment groups (Table 2). Patients receiving tofacitinib at either dose showed significant improvements from baseline in DAS28–3(CRP) versus placebo as early as week 2, which continued through the placebo-controlled period to month 3 in both studies (Additional file 1: Table S1). LS mean change from baseline in DAS28–3(CRP) continued to decrease with tofacitinib treatments through month 6 in both studies, and improvement was maintained until the end of the 12-month OPAL Broaden study (Additional file 1: Table S1). Patients who advanced from placebo to tofacitinib treatments at month 3 showed improvements from baseline in DAS28–3(CRP) and LS mean change from baseline in DAS28–3(CRP) similar to patients receiving tofacitinib 5 or 10 mg BID throughout the studies at month 12 in OPAL Broaden and month 6 in OPAL Beyond (Additional file 1: Table S1).

### Effect sizes and standardized response means

Based on the proposed levels of responsiveness, the effect sizes and standardized response means for all treatments across all composite endpoints were large (≥0.80). The effect size for the composite endpoints was highest for PASDAS across all treatment groups at months 3, 6, and 12 in OPAL Broaden and months 3 and 6 in OPAL Beyond and was lowest for DAPSA across treatments, studies, and time points, with the exception of the adalimumab group in OPAL Broaden at month 3, in which the effect size for both DAPSA and CPDAI was 1.05 (Table 3). Effect size increased with time on treatment across endpoints, studies, and treatment (Table 3). The effect size for all endpoints was higher in the OPAL Broaden study compared with OPAL Beyond at both months 3 and 6 with both tofacitinib doses, with the exception of CPDAI with tofacitinib 5 mg BID, which was lower (Table 3).

**Table 3:** Effect sizes and standardized response means across studies

	Effect size					Standardized response mean				
	OPAL Broaden			OPAL Beyond		OPAL Broaden			OPAL Beyond	
	Tofacitinib 5 mg BID *N* = 68	Tofacitinib 10 mg BID *N* = 62	Adalimumab 40 mg SC Q2W *N* = 66	Tofacitinib 5 mg BID *N* = 64	Tofacitinib 10 mg BID *N* = 62	Tofacitinib 5 mg BID *N* = 68	Tofacitinib 10 mg BID *N* = 62	Adalimumab 40 mg SC Q2W *N* = 66	Tofacitinib 5 mg BID *N* = 64	Tofacitinib 10 mg BID *N* = 62
Month 3										
PASDAS	1.73	2.40	1.69	1.53	1.84	1.42	1.75	1.73	1.26	1.53
DAPSA	0.90	1.23	1.05	0.81	0.84	1.05	1.25	1.47	0.94	1.15
CPDAI	1.03	1.53	1.05	1.41	1.45	0.89	1.27	1.11	1.11	1.49
DAS28–3(CRP)	1.47	1.77	1.37	1.07	1.16	1.25	1.46	1.50	1.14	1.29
Month 6										
PASDAS	2.17	2.81	1.98	1.88	2.10	1.76	2.11	1.64	1.49	1.74
DAPSA	1.15	1.43	1.24	0.97	0.92	1.18	1.34	1.43	1.11	0.94
CPDAI	1.53	1.88	1.44	1.65	1.75	1.52	1.52	1.25	1.23	1.55
DAS28–3(CRP)	1.93	2.11	1.68	1.34	1.23	1.47	1.68	1.79	1.43	1.28
Month 12										
PASDAS	2.51	3.05	2.07	–	–	2.10	2.21	1.60	–	–
DAPSA	1.50	1.57	1.30	–	–	1.60	1.41	1.36	–	–
CPDAI	1.95	2.12	1.59	–	–	1.59	1.64	1.33	–	–
DAS28–3(CRP)	2.25	2.18	1.77	–	–	1.69	1.65	1.71	–	–

*N* = number of patients in the full analysis set with baseline psoriasis body surface area affected greater than or equal to 3% and with no missing values for any of the composite endpoints at baseline and months 3, 6, and 12 (OPAL Broaden only for month 12). This subset of patients was used for the calculation of effect sizes and standardized response means

*Abbreviations*: *BID* twice daily, *CPDAI* Composite Psoriatic Disease Activity Index, *DAPSA* Disease Activity Index for Psoriatic Arthritis, *DAS28–3(CRP)* 3-component Disease Activity Score using 28 joints with C-reactive protein, *OPAL* Oral Psoriatic Arthritis triaL, *PASDAS* Psoriatic Arthritis Disease Activity Score, *Q2W* once every 2 weeks, *SC* subcutaneous

The highest standardized response mean was observed for PASDAS at months 3 and 6 in OPAL Beyond and at all time points with tofacitinib treatment at either dose in OPAL Broaden (Table 3). In OPAL Broaden, with adalimumab treatment, the highest standardized response mean was observed for PASDAS at month 3 and for DAS28–3(CRP) at months 6 and 12 (Table 3). In both OPAL Broaden and OPAL Beyond, the standardized response mean increased with time on treatment in patients receiving tofacitinib 5 mg BID for all endpoints, and for patients receiving tofacitinib 10 mg BID, with the exception of DAS28–3(CRP) and DAPSA at month 6 in OPAL Beyond (Table 3). The standardized response mean for PASDAS, DAS28–3(CRP), and DAPSA was higher in the OPAL Broaden study compared with OPAL Beyond at both months 3 and 6 with both tofacitinib doses (Table 3). For CPDAI, the standardized response mean was lower at month 3 in OPAL Broaden versus OPAL Beyond with both tofacitinib doses; at month 6, the standardized response mean was higher with tofacitinib 5 mg BID and lower with tofacitinib 10 mg BID in OPAL Broaden versus OPAL Beyond (Table 3).

### Outcomes stratified by MDA response and multiple regression analysis

**Fig. 3:**
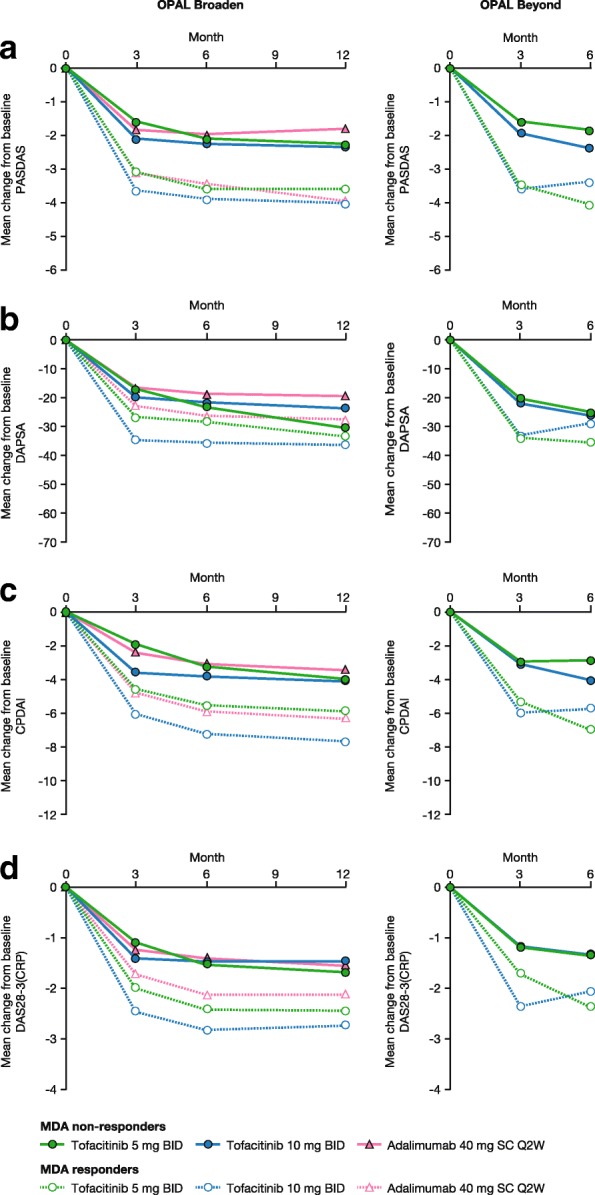
Change from baseline in composite endpoint scores by MDA response status across studies, **a** PASDAS, **b** DAPSA, **c** CPDAI, **d** DAS28-3 (CRP). For complete data, see Additional file 2: Table S2. MDA response was defined as five of the following seven criteria being met: TJC ≤1, SJC ≤1, Psoriasis Area and Severity Index score ≤1 or psoriasis BSA ≤3%, patient arthritis pain (VAS) ≤15 mm, patient’s global assessment of arthritis (VAS) ≤20 mm, HAQ-DI ≤0.5, tender entheseal points (using LEI) ≤1. Abbreviations: *BID* twice daily, *BSA* body surface area, *CPDAI* Composite Psoriatic Disease Activity Index, *DAPSA* Disease Activity Index for Psoriatic Arthritis, *DAS28–3(CRP)* 3-component Disease Activity Score using 28 joints with C-reactive protein, *FAS* full analysis set, *HAQ-DI* Health Assessment Questionnaire-Disability Index, *LEI* Leeds Enthesitis Index, *MDA* minimal disease activity, *OPAL* Oral Psoriatic Arthritis triaL, *PASDAS* Psoriatic Arthritis Disease Activity Score, *Q2W* once every 2 weeks, *SC* subcutaneous, *SJC* swollen joint count, *TJC* tender joint count, *VAS* visual analog scale

Mean changes from baseline appeared greater in MDA responders than MDA non-responders for all composite endpoints across all time points and treatments; however, no statistical comparison was made between these groups (Fig. 3 and Additional file 2: Table S2). Multiple logistic regression analysis of MDA response indicated that in both OPAL Broaden and OPAL Beyond there were statistically significant associations between change from baseline in PASDAS and MDA response at all time points in both studies for both doses of tofacitinib except month 3 for tofacitinib 10 mg BID (Table 4). For DAPSA, statistically significant associations were seen for tofacitinib 5 mg BID at 12 months in OPAL Broaden and at 6 months in OPAL Beyond. Significant associations were also observed for tofacitinib 10 mg BID at both 3 and 6 months in OPAL Beyond. In contrast, there were no statistically significant associations within CPDAI at any time point in either study. For comparison, statistically significant associations were noted for tofacitinib 10 mg BID at 6 and 12 months in OPAL Broaden and at 3 and 6 months in OPAL Beyond for DAS28–3(CRP).

**Table 4:** Comparison of associations of composite endpoints with MDA response across time points and studies

	OPAL Broaden						OPAL Beyond			
	Tofacitinib 5 mg BID *N* = 68		Tofacitinib 10 mg BID *N* = 62		Adalimumab 40 mg SC Q2W *N* = 66		Tofacitinib 5 mg BID *N* = 64		Tofacitinib 10 mg BID *N* = 62	
	STB	R	STB	R	STB	R	STB	R	STB	R
Month 3										
PASDAS	−0.73*	−0.19	−0.71	−0.13	−0.70*	−0.20	−1.23**	−0.26	−0.53	0.00
DAPSA	0.64	0.10	0.09	0.00	0.35	0.00	0.38	0.00	0.87*	0.23
CPDAI	−0.07	0.00	0.15	0.00	−0.33	−0.02	0.17	0.00	−0.71	−0.16
DAS28–3(CRP)	−0.58	−0.06	−0.34	0.00	−0.11	0.00	−0.08	0.00	−0.75*	−0.22
Month 6										
PASDAS	−0.91*	−0.20	−0.83*	−0.17	−0.61	−0.12	−1.27*	−0.21	−0.77*	−0.16
DAPSA	0.78	0.15	0.83	0.15	0.47	0.04	0.82*	0.21	1.00**	0.26
CPDAI	0.08	0.00	−0.00	0.00	−0.17	0.00	−0.48	0.00	0.04	0.00
DAS28–3(CRP)	−0.53	−0.08	−1.23**	−0.26	−0.42	−0.11	−0.44	0.00	−0.69*	−0.20
Month 12										
PASDAS	−1.02**	−0.29	−0.87*	−0.18	−1.88***	−0.35	–	–	–	–
DAPSA	0.79**	0.24	0.51	0.04	0.86	0.11	–	–	–	–
CPDAI	0.08	0.00	−0.09	0.00	−0.00	0.00	–	–	–	–
DAS28–3(CRP)	−0.68*	−0.20	−0.75*	−0.19	−0.18	0.00	–	–	–	–

*P≤0.05, **P<0.01, ***P<0.001 testing the null hypothesis that the slope coefficient is equal to 0, based on the Wald statistic from the multiple logistic regression model

*N* = number of patients included in the multiple logistic regression model

For a given time point and treatment group, a multiple logistic regression was performed on MDA evaluated at this time point as a dependent variable and the changes from baseline in the composite endpoints measured at the same time point as predictors. The slope coefficient for a composite endpoint from this regression model is standardized (STB) to permit comparison of the associations of these composite endpoints with the MDA response. An additional statistic, logistic pseudo partial correlation (R, range − 1 to 1), was also computed as a measure of correlation between the composite endpoints and MDA response [15]

*Abbreviations*: *BID* twice daily, *CPDAI* Composite Psoriatic Disease Activity Index, *DAPSA* Disease Activity Index for Psoriatic Arthritis, *DAS28–3(CRP)* 3-component Disease Activity Score using 28 joints with C-reactive protein, *MDA* minimal disease activity, *OPAL* Oral Psoriatic Arthritis triaL, *PASDAS* Psoriatic Arthritis Disease Activity Score, *Q2W* once every 2 weeks, *SC* subcutaneous

## Discussion

In the phase 3 studies OPAL Broaden and OPAL Beyond, patients with active PsA receiving tofacitinib 5 and 10 mg BID showed improvements versus placebo throughout the 3-month placebo-controlled period for the composite endpoints assessed. These improvements were subsequently maintained to month 6 in OPAL Beyond and month 12 in OPAL Broaden. Adalimumab had comparable efficacy to tofacitinib across the composite endpoints in OPAL Broaden.

OPAL Broaden and OPAL Beyond involved two distinct populations of patients with PsA: csDMARD-IR/TNFi-naïve patients in OPAL Broaden and TNFi-IR patients in OPAL Beyond. Despite the difference in patient populations, baseline values for the composite endpoints were broadly similar across studies and treatments. Generally, LS mean changes from baseline were greater, and the effect size and standardized response mean were higher, in the OPAL Broaden study compared with OPAL Beyond. This suggests that the TNFi-naïve patients in OPAL Broaden showed more marked treatment responses than the TNFi-IR patients in OPAL Beyond, similar to previous reports for PsA treatment [18–20].

PASDAS baseline scores in OPAL Broaden were comparable with values reported in an equivalent study population [3]; however, along with the PASDAS baseline scores in OPAL Beyond, they were somewhat higher than those reported in a study of standard care [21] and patients in clinical practice [22]. In the GRACE (GRAPPA Composite Exercise) study, designed to develop composite disease activity and responder measures for PsA, a mean score of 5.30 for PASDAS was reported for patients changing treatment and this was taken as a surrogate for high disease activity [11]. The mean baseline PASDAS levels reported in this study were therefore suggestive of high disease activity in both OPAL Broaden and OPAL Beyond, and following 3 months of treatment, PASDAS levels dropped below this threshold. In addition, the GRACE study defined a good response as a PASDAS score of less than or equal to 3.2, following a decrease in score of greater than or equal to 1.6 from baseline [17]; in this study, this was achieved at month 12 in OPAL Broaden by 44.2% and 47.5% of patients receiving tofacitinib 5 and 10 mg BID, respectively, and at month 6 in OPAL Beyond by 28.5% and 28.9% of patients receiving tofacitinib 5 and 10 mg BID, respectively. Of note, a PASDAS score of less than or equal to 3.2 has been defined as low disease activity [17] and less than or equal to 1.9 as very low disease activity [23].

OPAL Broaden DAPSA baseline scores were slightly lower than baseline scores in an equivalent study population [3] but higher than reported in clinical practice [24]. In the GRACE study, patients changing treatment (considered to have high disease activity) had a mean DAPSA score of 41.91 [11], suggesting that patients in OPAL Broaden and OPAL Beyond had high levels of disease activity. Indeed, in a recent study analyzing data from 30 patients with PsA in an observational database, the cutoff for a DAPSA score indicating high disease activity was greater than 28 [25]. In this study, mean DAPSA scores were below the high disease activity score reported in the GRACE study after 3 months of active treatment in all groups [11].

In contrast to the findings with the other composite measures, the baseline CPDAI scores reported for OPAL Broaden and OPAL Beyond were somewhat lower than mean CPDAI score of 11.65 reported for patients changing treatment (surrogate for high disease activity) in the GRACE study [11]; thus, CPDAI scores did not appear to indicate patients with high baseline disease activity in these patient populations. However, another study has suggested a high disease activity threshold of greater than 7 for CPDAI [26]; mean CPDAI scores were below this threshold after 3 months of active treatment across all groups and both studies.

The DAS28–3(CRP) was included for comparative purposes only. Baseline DAS28–3(CRP) scores were somewhat higher than the mean DAS28–3(CRP) score of 3.96 observed for patients changing treatment (a surrogate for high disease activity) in the GRACE study [11]; however, DAS28–3(CRP) scores in this study were reduced below this level following 3 months of treatment. It should be noted, however, that this measure was developed and validated for rheumatoid arthritis and there are several reasons why it is inappropriate as a composite measure for assessing PsA, particularly as it measures only articular outcomes and excludes joints of the foot and ankle, potentially missing important inflammatory disease [27].

All reported effect size and standardized response mean values were greater than 0.80, the value generally taken to indicate a large treatment effect or response [3]. The largest effect size was observed at all time points and treatments for the composite endpoint PASDAS; this is consistent with findings reported for golimumab [3]. Effect size and standardized response mean generally showed increases with time on treatment, indicating that the composite endpoints demonstrated time-dependent improvement, as might be expected. Analysis of the percentage of PASDAS responders over time also demonstrated the ability of the PASDAS instrument to detect treatment-related changes in PsA disease activity.

The definition of MDA using the criteria applied in this analysis and in previous tofacitinib publications [9, 10] has utility for identifying treatment response and as such may be used as a target to guide treatment decisions [16]. When the standardized slope coefficients of the composite endpoints (STBs) from a multiple logistic regression model were compared, the change in PASDAS had the largest magnitude of association with MDA response among all the composite endpoints examined, suggesting that it had the strongest predictive ability compared with DAPSA and CPDAI; CPDAI had the lowest predictive ability of the endpoints.

The differing findings with respect to tofacitinib treatment for the three disease-specific composite endpoints considered in this analysis could have resulted from the different composition of the endpoints evaluated. The PASDAS and CPDAI both include assessment of the skin manifestations of PsA (the PASDAS by inclusion of the patient’s global “arthritis and psoriasis” VAS) and the severity of enthesitis and dactylitis as well as TJC and SJC. DAPSA, however, is focused on TJC and SJC, with no consideration of skin disease, enthesitis, or dactylitis and an arthritis-focused global score. The PASDAS and CPDAI also both incorporate PROs; the PASDAS incorporates the PCS score of the SF-36v2 acute, and the CPDAI the DLQI and ASQoL. In this analysis, the PASDAS appeared to be the most sensitive to improvements in the signs and symptoms of PsA related to treatment with tofacitinib and adalimumab; the effect size observed with the PASDAS was higher than for any other endpoint at all time points in both studies. The ability of the PASDAS to detect change in these two studies might reflect the components of the measure; skin manifestations, enthesitis, dactylitis, and PROs all appeared to be sensitive to treatment-related changes in OPAL Broaden and OPAL Beyond, although the adoption of a hierarchical testing scheme for key secondary endpoints precluded demonstration of significance for all measures and time points [9, 10]. The CPDAI also incorporates skin, enthesitis, dactylitis, and PROs but appeared less sensitive to treatment differences than PASDAS though with generally higher effect size and standardized response mean than DAPSA. Inclusion of the axial disease domain in CPDAI (which does not feature in the other composite endpoints assessed) could offer an explanation as to why tofacitinib had the least impact on this composite; it may be that axial disease responds to a lesser extent than the other domains to treatment with tofacitinib and this may have impacted the final composite score. The CPDAI may also be less responsive because of the way it is constructed: the CPDAI is essentially a categorical measure re-expressed as a continuous scale and the hierarchical thresholds may blunt responsiveness. As previously discussed, the utility of DAS28–3(CRP) is limited because of the small number of components included in the composite and the lack of inclusion of measures of skin disease, enthesitis, dactylitis, or PROs.

It is clear from these analyses that PASDAS has superior performance in this context and it has already been reported that the consensus view is that PASDAS should be the outcome measure of choice in PsA clinical trials [28]. The DAPSA is easier to evaluate but there are arguments against this measure; PsA is a complex multifaceted disease which requires appropriate evaluation across domains, and measures such as the DAPSA, though easy to perform in practice, do not fulfill this function. In terms of clinical practice, the PASDAS does provide a challenge in both acquiring the data and processing the result: the first challenge represents the general case of clinical assessment in PsA; the second challenge is easily overcome by the use of predefined spreadsheets and web-based resources.

This analysis had a number of limitations. The OPAL Broaden and OPAL Beyond studies were not designed for evaluation of the composite endpoints’ longitudinal validity and sensitivity to change. In addition, for the calculation of effect size and standardized response mean, only patients with greater than or equal to 3% psoriasis BSA affected at baseline were included, with no missing values of the composite endpoints across multiple visits. Consequently, patient numbers were relatively low in some cases; CPDAI data were available for only 63% and 52% of patients receiving tofacitinib 10 mg BID in OPAL Broaden at month 12 and OPAL Beyond at month 6, respectively, and effect size and standardized response mean were calculated in only 47–64% of patients. Also, there was no adjustment for multiplicity; therefore, the *P* values reported for comparison with placebo should be considered nominal.

## Conclusions

Overall, while the merits of specific composite measures for assessing PsA status are under discussion [29], this evaluation of three different composite scales for the assessment of tofacitinib efficacy in PsA supports the use of sensitive composite measures, particularly PASDAS, for the evaluation of efficacy of treatments that impact multiple PsA disease domains.

## Additional files

Additional file 1: **Table S1.** Least squares (LS) mean change from baseline in composite endpoint scores (full analysis set, or FAS). (DOCX 44 kb)

Additional file 2: **Table S2.** Change from baseline in composite endpoint scores by minimal disease activity (MDA) response status across studies. (DOCX 39 kb)

## Abbreviations

ACR20: Greater than or equal to 20% improvement according to the criteria of the American College of Rheumatology
ASQoL: Ankylosing Spondylitis Quality of Life
BID: Twice daily
BSA: Body surface area
CPDAI: Composite Psoriatic Disease Activity Index
CRP: C-reactive protein
csDMARD: Conventional synthetic disease-modifying anti-rheumatic drug
DAPSA: Disease Activity Index for Psoriatic Arthritis
DAS28–3(CRP): 3-component Disease Activity Score using 28 joints with C-reactive protein
DLQI: Dermatology Life Quality Index
FAS: Full analysis set
GRACE: GRAPPA Composite Exercise
GRAPPA: Group for Research and Assessment of Psoriasis and Psoriatic Arthritis
HAQ-DI: Health Assessment Questionnaire-Disability Index
IR: Inadequate responders
JAK: Janus kinase
LEI: Leeds Enthesitis Index
LS: Least squares
MDA: Minimal disease activity
OPAL: Oral Psoriatic Arthritis triaL
PASDAS: Psoriatic Arthritis Disease Activity Score
PASI: Psoriasis Area and Severity Index
PCS: Physical component summary
PRO: Patient-reported outcome
PsA: Psoriatic arthritis
Q2W: Once every 2 weeks
SC: Subcutaneous
SD: Standard deviation
SE: Standard error
SF-36v2: 36-item short-form survey version 2
SJC: Swollen joint count
TJC: Tender joint count
TNFi: Tumor necrosis factor inhibitor
VAS: Visual analog scale
