# Supplementary material for: Disease-specific composite measures for psoriatic arthritis are highly responsive to a Janus kinase inhibitor treatment that targets multiple domains of disease
Source: Arthritis Res Ther. 2018 Oct 29;20:242. doi: 10.1186/s13075-018-1739-0 (PMC6235208; doi:10.1186/s13075-018-1739-0)
Supplement: Supplementary file 1 — Table S1. Least squares (LS) mean change from baseline in composite endpoint scores (full analysis set, or FAS). (DOCX 44 kb) [file 13075_2018_1739_MOESM1_ESM.docx]

**Additional File 1**

**Supplementary Table** LS mean change from baseline in composite endpoint scores (FAS)

|  | **LS mean change from baseline in composite endpoint scores (SE) [number of patients evaluable at time point]** | | | | | | | | |
| --- | --- | --- | --- | --- | --- | --- | --- | --- | --- |
|  | **OPAL Broaden (*N* = 422)** | | | | | **OPAL Beyond (*N* = 394)** | | | |
|  | Tofacitinib  5 mg BID *N* = 107 | Tofacitinib  10 mg BID *N* = 104 | Adalimumab 40 mg SC Q2W *N* = 106 | Placebo -> tofacitinib  5 mg BID *N* = 52^a^ | Placebo -> tofacitinib  10 mg BID *N* = 53^a^ | Tofacitinib  5 mg BID *N* = 131 | Tofacitinib  10 mg BID *N* = 132 | Placebo -> tofacitinib  5 mg BID *N* = 66^a^ | Placebo -> tofacitinib  10 mg BID *N* = 65^a^ |
| **PASDAS** | | | | | | | | | |
| Month 1 | -1.25 (0.12) [101]*** | -1.62 (0.12) [101]*** | -1.31 (0.12) [104]*** | -0.68 (0.13) [99] | | -1.42 (0.11) [120]*** | -1.64 (0.11) [120]*** | -0.61 (0.10) [124] | |
| Month 3 | -1.99 (0.14) [97]*** | -2.39 (0.14) [101]*** | -2.17 (0.14) [98]*** | -1.21 (0.15) [99] | | -1.93 (0.14) [118]*** | -2.14 (0.14) [112]*** | -0.83 (0.14) [114] | |
| Month 6 | -2.45 (0.14) [98] | -2.65 (0.15) [97] | -2.55 (0.15) [98] | -2.17 (0.21) [47] | -2.39 (0.21) [47] | -2.28 (0.14) [111] | -2.41 (0.14) [106] | -2.11 (0.20) [52] | -2.37 (0.20) [55] |
| Month 9 | -2.64 (0.15) [96] | -2.84 (0.15) [94] | -2.75 (0.15) [94] | -2.43 (0.21) [45] | -2.98 (0.21) [45] | - | - | - | - |
| Month 12 | -2.78 (0.14) [93] | -2.95 (0.14) [93] | -2.68 (0.14) [93] | -2.54 (0.20) [43] | -2.87 (0.21) [42] | - | - | - | - |
| **DAPSA** | | | | | | | | | |
| Week 2 | -8.55 (1.27) [105] | -12.49 (1.29) [101]*** | -9.87 (1.32) [101]* | -5.97 (1.40) [99] | | -11.42 (1.31) [124]** | -10.56 (1.30) [130]** | -5.60 (1.30) [127] | |
| Month 1 | -12.02 (1.47) [105] | -17.37 (1.49) [102]*** | -13.57 (1.52) [103]* | -8.58 (1.59) [103] | | -15.14 (1.38) [126]*** | -14.85 (1.38) [129]*** | -8.40 (1.37) [129] | |
| Month 2 | -18.04 (1.59) [101]** | -22.77 (1.60) [102]*** | -18.27 (1.64) [99]** | -12.42 (1.70) [101] | | -20.65 (1.67) [123]*** | -19.11 (1.67) [126]*** | -8.71 (1.69) [118] | |
| Month 3 | -20.20 (1.72) [101]** | -24.40 (1.73) [103]*** | -19.30 (1.77) [98]* | -13.79 (1.82) [101] | | -22.46 (1.67) [123]*** | -21.04 (1.70) [117]*** | -8.60 (1.69) [117] | |
| Month 4 | -23.12 (1.65) [101] | -24.26 (1.67) [99] | -21.18 (1.69) [100] | -20.54 (2.35) [50] | -20.82 (2.37) [50] | -24.80 (1.65) [124] | -22.71 (1.66) [121] | -19.62 (2.40) [55] | -22.25 (2.38) [57] |
| Month 6 | -23.90 (1.68) [100] | -27.66 (1.70) [99] | -24.00 (1.73) [98] | -25.83 (2.40) [48] | -24.39 (2.42) [48] | -26.00 (1.74) [122] | -21.58 (1.78) [113] | -22.86 (2.54) [56] | -21.50 (2.54) [55] |
| Month 9 | -28.54 (1.60) [98] | -29.16 (1.62) [96] | -25.31 (1.65) [95] | -27.76 (2.28) [47] | -30.06 (2.31) [46] | - | - | - | - |
| Month 12 | -30.18 (1.60) [95] | -30.00 (1.61) [96] | -24.82 (1.65) [92] | -28.81 (2.29) [44] | -29.76 (2.32) [44] | - | - | - | - |
| **CPDAI^b^** | | | | | | | | | |
| Month 1 | -1.9 (0.28) [79] | -2.3 (0.31) [67]** | -1.5 (0.30) [74] | -1.2 (0.31) [78] | | -2.3 (0.25) [78]*** | -2.3 (0.25) [76]*** | -0.8 (0.24) [81] | |
| Month 3 | -2.9 (0.34) [76] | -4.2 (0.36) [67]*** | -3.1 (0.34) [74]* | -2.2 (0.36) [78] | | -3.3 (0.31) [70]*** | -3.4 (0.31) [73]*** | -1.6 (0.31) [72] | |
| Month 6 | -4.0 (0.35) [75] | -5.1 (0.38) [65] | -4.3 (0.36) [73] | -3.7 (0.48) [39] | -3.9 (0.52) [34] | -3.9 (0.33) [73] | -4.1 (0.34) [68] | -3.7 (0.49) [31] | -3.6 (0.46) [35] |
| Month 9 | -4.4 (0.36) [73] | -5.4 (0.38) [64] | -4.7 (0.37) [70] | -4.5 (0.49) [38] | -5.3 (0.53) [31] | - | - | - | - |
| Month 12 | -5.1 (0.36) ([71] | -5.7 (0.39) [63] | -4.7 (0.37) [70] | -4.7 (0.50) [36] | -5.0 (0.54) [31] | - | - | - | - |
| **DAS28-3(CRP)** | | | | | | | | | |
| Week 2 | -0.70 (0.07) [105]*** | -0.87 (0.07) [101]*** | -0.80 (0.07) [102]*** | -0.28 (0.08) [99] | | -0.71 (0.07) [125]*** | -0.66 (0.07) [130]*** | -0.29 (0.07) [127] | |
| Month 1 | -0.91 (0.09) [105]*** | -1.28 (0.09) [103]*** | -1.04 (0.09) [104]*** | -0.41 (0.09) [103] | | -0.87 (0.08) [127]** | -0.95 (0.08) [129]*** | -0.52 (0.08) [129] | |
| Month 2 | -1.09 (0.09) [102]*** | -1.43 (0.09) [102]*** | -1.41 (0.09) [100]*** | -0.68 (0.10) [101] | | -1.28 (0.09) [125]*** | -1.20 (0.09) [126]*** | -0.65 (0.09) [120] | |
| Month 3 | -1.33 (0.10) [101]*** | -1.63 (0.10) [103]*** | -1.51 (0.10) [99]*** | -0.77 (0.11) [101] | | -1.38 (0.10) [123]*** | -1.23 (0.10) [118]*** | -0.61 (0.10) [117] | |
| Month 4 | -1.48 (0.11) [101] | -1.71 (0.11) [99] | -1.65 (0.11) [101] | -1.41 (0.15) [50] | -1.40 (0.15) [50] | -1.47 (0.10) [125] | -1.38 (0.10) [121] | -1.27 (0.14) [55] | -1.47 (0.14) [57] |
| Month 6 | -1.66 (0.11) [100] | -1.94 (0.11) [99] | -1.81 (0.11) [99] | -1.72 (0.15) [48] | -1.62 (0.15) [48] | -1.57 (0.10) [123] | -1.35 (0.10) [113] | -1.47 (0.14) [56] | -1.51 (0.14) [55] |
| Month 9 | -1.82 (0.11) [99] | -1.93 (0.11) [96] | -1.89 (0.11) [96] | -1.93 (0.16) [47] | -2.05 (0.16) [46] | - | - | - | - |
| Month 12 | -1.93 (0.11) [95] | -2.05 (0.11) [96] | -1.91 (0.11) [93] | -2.02 (0.16) [44] | -1.97 (0.16) [44] | - | - | - | - |

**p* ≤ 0.05, ***p* < 0.01, ****p* < 0.001 vs placebo

^a^For visits up to month 3, patients from the two placebo sequences were combined into a single placebo group

^b^Only patients with baseline psoriasis BSA ≥3% were included

*N* = number of patients in the FAS

Change from baseline analyses were based on a repeated measures model, without imputation for missing values in the FAS, with the fixed effects of treatment, visit, treatment-by-visit interaction, geographic location, and baseline value; an unstructured covariance matrix was used. For results up to month 3, patients randomized to the two placebo sequences were combined into a single placebo group. The repeated measures model included data of all visits up to month 3 for the treatment groups of tofacitinib 5 mg BID, tofacitinib 10 mg BID, adalimumab 40 mg SC Q2W (OPAL Broaden only), and placebo. For results beyond month 3 to the end of study, the two placebo sequences were analyzed separately, reporting only results after month 3. *P* values are not reported after month 3 as the placebo-controlled period ended at month 3

*BID* twice daily, *BSA* body surface area, *CPDAI* Composite Psoriatic Disease Activity Index, *DAPSA* Disease Activity Index for Psoriatic Arthritis, *DAS28-3(CRP)* 3-component Disease Activity Score using 28 joints with C-reactive protein, *FAS* full analysis set, *LS* least squares, *OPAL* Oral Psoriatic Arthritis triaL, *PASDAS* Psoriatic Arthritis Disease Activity Score, *Q2W* once every 2 weeks, *SC* subcutaneous, *SE* standard error
