# Supplementary material for: Disease-specific composite measures for psoriatic arthritis are highly responsive to a Janus kinase inhibitor treatment that targets multiple domains of disease
Source: Arthritis Res Ther. 2018 Oct 29;20:242. doi: 10.1186/s13075-018-1739-0 (PMC6235208; doi:10.1186/s13075-018-1739-0)
Supplement: Supplementary file 2 — Table S2. Change from baseline in composite endpoint scores by minimal disease activity (MDA) response status across studies. (DOCX 39 kb) [file 13075_2018_1739_MOESM2_ESM.docx]

**Additional File 2**

**Supplementary Table** Change from baseline in composite endpoint scores by MDA response status across studies

|  | **Mean change from baseline (SD)** | | | | | | | | | |
| --- | --- | --- | --- | --- | --- | --- | --- | --- | --- | --- |
|  | **OPAL Broaden** | | | | | | **OPAL Beyond** | | | |
|  | **MDA non-responders** | | | **MDA responders** | | | **MDA non-responders** | | **MDA responders** | |
|  | Tofacitinib  5 mg BID | Tofacitinib 10 mg BID | Adalimumab 40 mg SC Q2W | Tofacitinib  5 mg BID | Tofacitinib 10 mg BID | Adalimumab 40 mg SC Q2W | Tofacitinib  5 mg BID | Tofacitinib 10 mg BID | Tofacitinib  5 mg BID | Tofacitinib 10 mg BID |
| **Month 3** | *N* = 53 | *N* = 49 | *N* = 49 | *N* = 15 | *N* = 13 | *N* = 17 | *N* = 49 | *N* = 47 | *N* = 15 | *N* = 15 |
| PASDAS | -1.59 (1.23) | -2.10 (1.17) | -1.83 (1.03) | -3.07 (1.11) | -3.63 (1.52) | -3.10 (1.37) | -1.60 (1.50) | -1.93 (1.30) | -3.45 (1.09) | -3.59 (1.52) |
| DAPSA | -16.84 (19.28) | -19.66 (14.34) | -16.58 (12.61) | -26.65 (10.68) | -34.59 (26.07) | -22.61 (10.68) | -20.01 (22.61) | -21.76 (17.30) | -33.88 (29.14) | -33.01 (29.67) |
| CPDAI | -1.91 (2.58) | -3.59 (2.99) | -2.41 (2.26) | -4.60 (2.56) | -6.08 (3.50) | -4.82 (3.19) | -2.96 (3.14) | -3.09 (2.19) | -5.33 (2.53) | -6.00 (2.36) |
| DAS28-3(CRP) | -1.10 (1.02) | -1.42 (1.03) | -1.25 (0.86) | -2.00 (0.83) | -2.47 (1.11) | -1.72 (1.01) | -1.19 (1.07) | -1.17 (0.98) | -1.71 (1.34) | -2.35 (1.14) |
| **Month 6** | *N* = 54 | *N* = 40 | *N* = 41 | *N* = 14 | *N* = 22 | *N* = 25 | *N* = 45 | *N* = 44 | *N* = 19 | *N* = 18 |
| PASDAS | -2.10 (1.20) | -2.25 (0.88) | -1.98 (1.23) | -3.59 (1.38) | -3.89 (1.41) | -3.42 (1.60) | -1.85 (1.39) | -2.38 (1.60) | -4.04 (1.25) | -3.38 (1.09) |
| DAPSA | -23.16 (21.74) | -21.58 (17.02) | -18.56 (16.33) | -28.14 (14.18) | -35.63 (21.75) | -26.26 (11.38) | -24.89 (23.32) | -26.01 (31.52) | -35.37 (28.85) | -28.78 (20.13) |
| CPDAI | -3.24 (2.21) | -3.83 (2.36) | -3.05 (2.57) | -5.57 (2.53) | -7.27 (3.69) | -5.92 (3.63) | -2.89 (2.70) | -4.07 (2.91) | -7.00 (2.98) | -5.72 (2.70) |
| DAS28-3(CRP) | -1.53 (1.15) | -1.48 (1.02) | -1.41 (0.91) | -2.42 (0.97) | -2.84 (0.88) | -2.14 (0.82) | -1.35 (1.01) | -1.33 (1.25) | -2.37 (1.17) | -2.06 (0.92) |
| **Month 12** | *N* = 41 | *N* = 35 | *N* = 40 | *N* = 27 | *N* = 27 | *N* = 26 | - | - | - | - |
| PASDAS | -2.26 (1.20) | -2.34 (0.86) | -1.81 (1.28) | -3.58 (1.09) | -4.02 (1.39) | -3.93 (1.29) | - | - | - | - |
| DAPSA | -30.37 (21.25) | -23.53 (19.53) | -19.24 (18.22) | -33.26 (17.30) | -36.14 (20.02) | -27.61 (12.54) | - | - | - | - |
| CPDAI | -4.00 (3.14) | -4.11 (2.93) | -3.43 (2.93) | -5.89 (2.34) | -7.70 (3.07) | -6.35 (3.50) | - | - | - | - |
| DAS28-3(CRP) | -1.69 (1.15) | -1.47 (1.08) | -1.55 (1.09) | -2.46 (1.10) | -2.74 (1.03) | -2.13 (0.87) | - | - | - | - |

*N* = Number of patients by MDA response status and treatment group at time point. Data are reported for patients in the FAS with baseline psoriasis BSA affected ≥3% and with no missing values for MDA or any of the composite endpoints at baseline, months 3, 6, and 12 (OPAL Broaden only for month 12) for tofacitinib and adalimumab groups

MDA response was defined as five of the following seven criteria being met: TJC ≤1, SJC ≤1, Psoriasis Area and Severity Index score ≤1 or psoriasis BSA ≤3%, patient arthritis pain (VAS) ≤15 mm, patient’s global assessment of arthritis (VAS) ≤20 mm, HAQ-DI ≤0.5, tender entheseal points (using LEI) ≤1

*BID* twice daily, *BSA* body surface area, *CPDAI* Composite Psoriatic Disease Activity Index, *DAPSA* Disease Activity Index for Psoriatic Arthritis, *DAS28-3(CRP)* 3-component Disease Activity Score using 28 joints with C-reactive protein, *FAS* full analysis set, *HAQ-DI* Health Assessment Questionnaire-Disability Index, *LEI* Leeds Enthesitis Index, *MDA* minimal disease activity, *OPAL* Oral Psoriatic Arthritis triaL, *PASDAS* Psoriatic Arthritis Disease Activity Score, *Q2W* once every 2 weeks, *SC* subcutaneous, *SD* standard deviation, *SJC* swollen joint count, *TJC* tender joint count, *VAS* visual analog scale
